# Networks in a Large-Scale Phylogenetic Analysis: Reconstructing Evolutionary History of Asparagales (Lilianae) Based on Four Plastid Genes

**DOI:** 10.1371/journal.pone.0059472

**Published:** 2013-03-18

**Authors:** Shichao Chen, Dong-Kap Kim, Mark W. Chase, Joo-Hwan Kim

**Affiliations:** 1 College of Life Science and Technology, Tongji University, Shanghai, China; 2 Division of Forest Resource Conservation, Korea National Arboretum, Pocheon, Gyeonggi-do, Korea; 3 Jodrell Laboratory, Royal Botanic Gardens, Kew, Richmond, United Kingdom; 4 Department of Life Science, Gachon University, Seongnam, Gyeonggi-do, Korea; BiK-F Biodiversity and Climate Research Center, Germany

## Abstract

Phylogenetic analysis aims to produce a bifurcating tree, which disregards conflicting signals and displays only those that are present in a large proportion of the data. However, any character (or tree) conflict in a dataset allows the exploration of support for various evolutionary hypotheses. Although data-display network approaches exist, biologists cannot easily and routinely use them to compute rooted phylogenetic networks on real datasets containing hundreds of taxa. Here, we constructed an original neighbour-net for a large dataset of Asparagales to highlight the aspects of the resulting network that will be important for interpreting phylogeny. The analyses were largely conducted with new data collected for the same loci as in previous studies, but from different species accessions and greater sampling in many cases than in published analyses. The network tree summarised the majority data pattern in the characters of plastid sequences before tree building, which largely confirmed the currently recognised phylogenetic relationships. Most conflicting signals are at the base of each group along the Asparagales backbone, which helps us to establish the expectancy and advance our understanding of some difficult taxa relationships and their phylogeny. The network method should play a greater role in phylogenetic analyses than it has in the past. To advance the understanding of evolutionary history of the largest order of monocots Asparagales, absolute diversification times were estimated for family-level clades using relaxed molecular clock analyses.

## Introduction

The only figure in *On the Origin of Species* [1] is an evolutionary tree that reflects Darwin’s vision of descent with modification from a common ancestor. Today, phylogenetic methods, or “tree-thinking” [2], form the foundation of inferences in evolutionary biology [3]–[5]. Bifurcating phylogenetic trees underlie our understanding of organismal evolution and are also proving instrumental in the development of a more robust classification system based on natural (evolutionary) relationships. Nevertheless, searches to determine “the tree” remain problematic, as they can often overlook conflicts in the dataset. Competing signals may arise from stochastic substitution processes, poorly fitting evolutionary models or the heuristic nature of many tree search algorithms. They may also be the result of hybridisation (including introgression), recombination, horizontal/lateral gene transfer, genome fusion, ancestral polymorphism/deep coalescence/incomplete lineage sorting and gene duplication-loss [6]. The detection of data conflicts, and the extent to which they affect analysis, becomes an important first step in phylogenetic analysis. Data-display networks may reveal reticulation patterns that are unsuspected in the data and that may have an important bearing on subsequent analyses and their interpretation. Unfortunately, this field is rather poorly developed at present [6], [7], and no tools are available that biologists can easily and consistently use on real data [8].

A neighbour net [9] is a split network that visualises certain collections of splits that have been derived from a distance matrix. These splits are constructed in an iterative fashion using a criterion similar to that used in the neighbour-joining (NJ) algorithm for tree construction [6], [10]. Morrison [6] reanalysed a dozen published datasets using split networks, highlighting aspects of the resulting network that could be important for interpretation of the phylogenetic tree and pointed out that the network method should play a greater role in phylogenetic analyses than it has in the past.

Asparagales is the largest order of monocots [11]–[16] with ca. 25,000–42,000 species (ca. 50% of monocots, or 10–15% of flowering plants), including important crop plants such as *Allium*, *Asparagus* and *Vanilla*, and a host of ornamentals such as irises, hyacinths and orchids [17]. The circumscription of Asparagales and the included families have undergone a series of changes in recent years. When the Angiosperm Phylogeny Group (APG) [18] was being formulated, numerous narrow circumscriptions for the families of Asparagales largely followed those of Dahlgren et al. [19], but it was noted (APG II, 2003) that broader circumscriptions were also possible, leading to a set of *sensu lato* (*s*.*l*.) families being proposed with the earlier set of *sensu stricto* families listed in brackets. In APG III [20], the number of families in Asparagales recognised fell from 26 [18] to 14 due to the elimination of these bracketed families. Furthermore, a set of subfamilies for the expanded asparagalean families was also published to be more manageable for teaching purposes and to facilitate communication among specialists [21]. A number of studies have sampled all/most families of Asparagales *sensu* APG [11], [14], [17], [18], [22]–[28], which have generally clarified the relationships among the families within Asparagales. However, uncertainties remain in two parts of the Asparagales phylogenetic tree. First, the exact relationships of some small families (e.g. Boryaceae, Doryanthaceae, Ixioliriaceae) in lower Asparagales and Aphyllanthoideae, in higher Asparagales, remain unresolved [17], [22], [23]. Previous studies [17], [22] found weak support for a sister relationship between Ixioliriaceae and Tecophilaeaceae, which in turn formed a polytomy or weakly supported sister group to Doryanthaceae. An analysis of morphological data, however, placed *Doryanthes* as sister to Iridaceae [24]. The position of Boryaceae also remains unclear relative to the rest of the families (except for the orchids) and the hypoxid clade [15], [23]. The positions of all of these families require additional evidence to establish their interrelationships [15]. Fay et al. [22] demonstrated that *Aphyllanthes* (monotypic, Aphyllanthoideae) has a destabilising position within Asparagaceae *s*.*l*. Other studies found that incompatible patterns were produced when analyzing different genes [14], [17]. The second problem, related to the extreme species richness, diverse morphology and complex taxonomic history of Asparagales, is that the sampling of taxa in previous studies has been limited, and many genera have not been included. Although it is clear that adding multigene sequences and sampling will produce a better hypothesis of evolutionary history, more incompatibilities could arise. Previous studies have demonstrated that bifurcating phylogenetic trees can be valuable tools for investigating the evolutionary history of Asparagales, but it is not possible to simultaneously display contradictory evolutionary signals on any such tree. Phylogenetic networks can provide a useful alternative means of analysis because they allow visualisation of competing evolutionary scenarios within a single figure [6], [29]. Here, we used a phylogenetic network method, neighbour net, to reanalyze the evolutionary history of Asparagales using a new comprehensive sampling of taxa and genes. In addition, using our estimates of the time of origin, we discuss their possible evolutionary history to improve our understanding of the processes that have generated such high diversity on this branch of the tree of life.

## Results

### Neighbour-net Pattern of the Data

**Figure 1:**
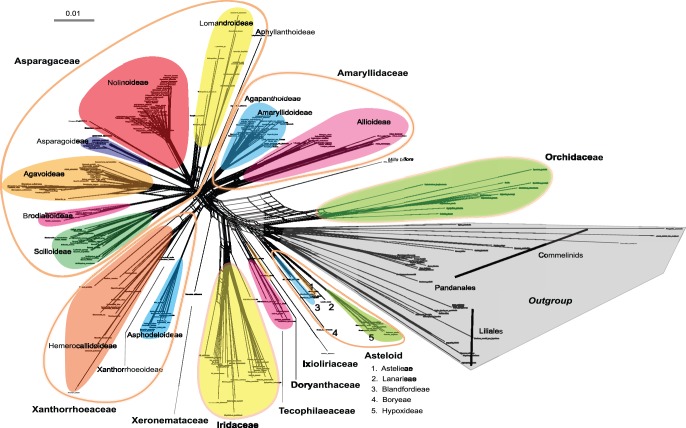
Neighbour net for Asparagales and outgroups. Neighbour net for Asparagales and outgroups with uncorrected p-distances, based on 284 species using four plastid genes: *atpB*, *matK*, *ndhF*, and *rbcL*. Families and subfamilies circumscriptions follow APG III (2009) and Chase et al. (2009) are colour-coded. Scale bar, 0.01.

**Figure 2:**
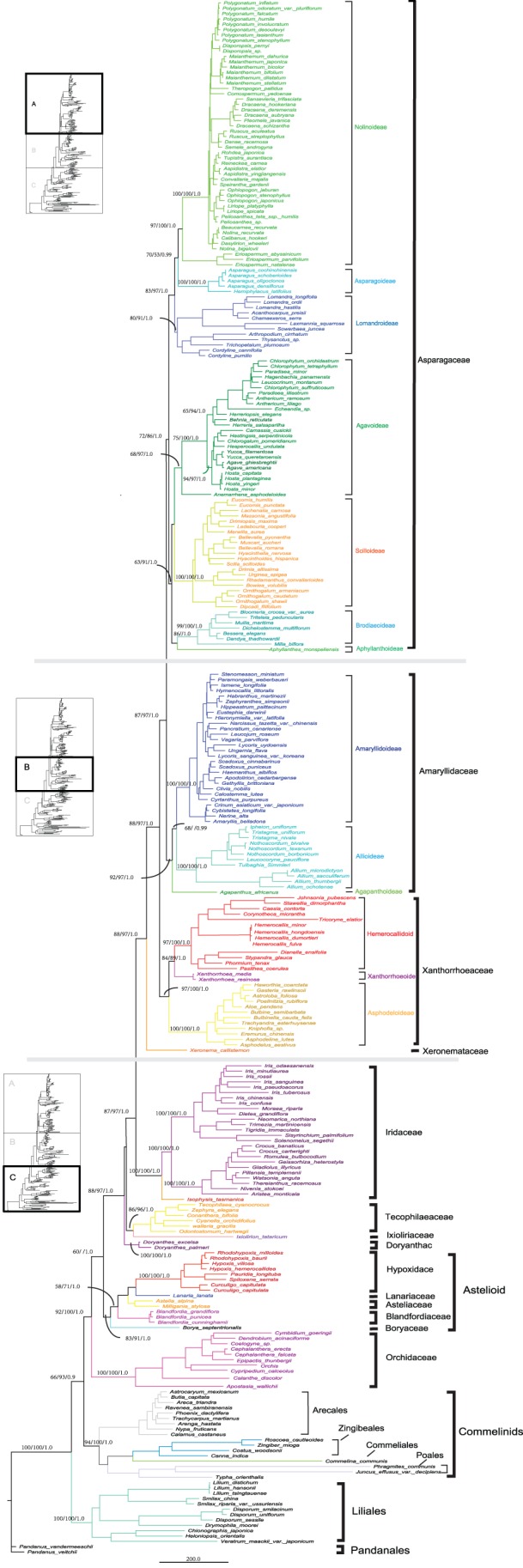
Consensus tree from Bayesian analysis of the four combined cpDNA datasets. The 50% majority rule consensus phylogram from partitioned Bayesian analysis of a combined matrix of 284 accessions and 6699 bp from four plastid genes: *atpB*, *matK*, *ndhF* and *rbcL*. The 400,000 generations before the point when the SDSF permanently fell below 0.01 (0.0016 at termination) were discarded as burn-in. Three types of support (bootstrap percentages for parsimony analyses with equal weights [EW]/successive approximations weighting [SW]/posterior probabilities for Bayesian analysis [PP]) are indicated on each branch. Major clades are named following the subfamily classification of three expanded asparagalean families proposed by Chase et al. (2009) and APG III (2009). The tree is subdivided as follows: **part A**, Asparagaceae and subfamilies; **part B**, Amaryllidaceae and Xanthorrhoeaceae and their subfamilies plus Xeronemataceae; **part C**, the basal nodes of Asparagales and outgroups (non-Asparagales taxa).

To gain a better understanding how conflicting signals were contained in the datasets, we constructed a neighbour net for the combined matrix of the four plastid genes (Figure 1), in which indels were not considered as informative characters. The outgroup *Pandanus* consisting of two species (Pandanales), together with Commelinales and Liliales species, were included as they are closely related to Asparagales [26]. The centre of the neighbour net was slightly netted, implying that the data support many conflicting deep splits. Nonetheless, the clades identified appeared to be quite robust as 21 clades were generally recovered, as indicated by the colours and arc labelling in Figure 1. The neighbour net showed strong support for monophyletic Asparagales. Commelinales, Liliales and Pandanales formed a close clade as the outgroup of Asparagales. The network largely confirmed the current recognised phylogenetic relationships [14], [22], [28]. In addition, there were strongly supported splits (and clusters), corresponding largely to the well-supported clades in the topology of the combined tree obtained with our parsimony and Bayesian analyses (Figure 2), except *Milla biflora*, which netted with Orchidaceae. Furthermore, most of the difficult taxon, with conflict position or extremely low resolution from regular phylogenetic analyses, appeared in critical state on the network graph. For example, Orchidaceae competed with Boryaceae and Blandfordiaceae etc. to root of Asparagales in previously researches [12], [28], [30]–[32].

### Phylogenetic Relationships

The total aligned matrix had 6,862 characters with 3,122 potentially phylogenetically informative sites for the four plastid genes: 1,472 base pairs (bp) for *atpB*, 1,820 bp for *matK*, 2,234 bp for *ndhF* and 1,336 bp for *rbcL*. In total, 163 base pairs were excluded from the combined matrix (1–17, 1449–1472, 3292–3316, 5480–5560, 6847–6862 bp), either at the beginning or end of sequences or where alignment of the *ndhF* sequences was ambiguous. Of the included characters, the numbers of potentially parsimony informative characters were 499 (33.9%) for *atpB*, 1,123 (61.7%) for *matK*, 1,160 (34.7%) for *ndhF* and 437 (32.7%) for *rbcL* (Table 2). The *matK* gene was the most variable among the four genes, but gave slightly fewer parsimony informative sites than *ndhF* due to the longer length of the latter. The *rbcL* gene was length-conserved with no gaps, and *atp*B had only few insertions/deletions (indels), whereas *matK* and *ndhF* included a number of indels.

**Table 2:** Statistics for the four genes analysed in this study.

Characters	*atpB*	*matK*	*ndhF*	*rbcL*	Combined
Aligned (bp)	1472	1820	2234	1336	6862
Included (bp)	1431	1819	2163	1286	6699
Parsimony uninformative	144	216	298	144	767
Parsimony informative	499	1123	1160	437	3122
Constant	829	481	776	755	2810
Transition/Transversion	2.58	1.72	2.57	3.16	2.18
G+C (%)	42.5	31.8	37.2	35.4	38.2
Tree length	26510	8275	9192	3269	24168
CI	0.248	0.295	0.275	0.258	0.272
RI	0.713	0.766	0.755	0.735	0.747
Variant rate (%)	33.9	61.7	34.7	32.7	45.5

Parsimony analyses of the individual plastid genes gave similar topologies as expected because these genes are inherited on the same linkage group. *Aphyllanthes* L. has previously been discussed as a problem taxon because of its labile phylogenetic position according to the analyses by different genes [17], [22]. As in previous analyses, we also performed analyses that excluded and included *Aphyllanthes*, which only affected position and support values in Asparagaceae *s*.*l*. Here we present the results found when *Aphyllanthes* was included.

The combined data Fitch analysis with equal weights (EW) produced 14,523 equally most-parsimonious trees of 24,168 steps, with a consistency index (CI, including autapomorphies) of 0.27 and a retention index (RI) of 0.75. With successive weights (SW), the number of equally most parsimonious trees was reduced to one (CI = 0.70, RI = 0.85). The SW tree is one of the trees found with Fitch weights. The Bayesian tree shows the PPs summarised from the set of recovered post-burn-in trees. The parameters of the GTR+I+G model used in this analysis are listed in Table 2. There was only one minor area of discordance between the maximum parsimony (MP) and Bayesian trees: the interrelationships among three families: Aphyllanthaceae, Themidaceae and Doryanthaceae.

Due to the similarity in topology of the strict consensus parsimonious tree and the Bayesian tree, the latter having higher resolution, only the Bayesian tree found in the combined analysis is shown in Figure 2. We report three kinds of support value: parsimony bootstrap percentages with EW, SW and PP for Bayesian analysis. Pandanales was the nominated outgroup in accordance with the results of previous studies [17], [22]. Within Asparagales, SW analysis had more nodes with strong support than EW, and the PP offered strong support for most nodes on the phylogenetic tree (Figure 2).

Asparagales *sensu* APG (1998) was monophyletic with strong support (92/100/1.0) as sister to the commelinids clade (66/93/0.9). A multiordinal clade, the commelinids monocots as a whole (Arecales, Commelinales-Zingiberales, Poales), was also strongly supported (94/100/1.0). A clade comprising Asparagales and Commelinids was grouped into a sister relationship with the Liliales clade (100/100/1.0). As in previous analyses, the order Asparagales can be divided into higher and lower asparagoid clades (*sensu* Chase et al. 1995a). However, this concept was recently replaced by that of core and non-core asparagoids [26], [33]. The core asparagoids formed a strongly supported monophyletic group containing two well-resolved clades, Asparagaceae *s*.*l*. (72/86/1.0) and Amaryllidaceae *s*.*l*. (92/97/1.0), which was recognised in APG III (2009). The Asparagaceae *s*.*l*. included a number of subfamilies represented by two clades, which was recognised in APG III (2009). The first clade (83/97/1.0) had Lomandroideae as sister to a monophyletic group (70/53/0.99) that consisted of Asparagoideae and Nolinoideae. The second clade (63/91/1.0) consisted of four subfamilies: Agavoideae, Scilloideae, Brodiaeoideae and Aphyllanthoideae. The result also suggested that the family Amaryllidaceae *s*.*l*. had two clades: (Amaryllidoideae+Allioideae) and Agapanthoideae. The core asparagoid clade was sister (88/97/1.0) to a strongly supported (97/100/1.0) family Xanthorrhoeaceae *s*.*l*. (*sensu* APG III), which included three subfamily clades: Asphodeloideae, Xanthorrhoeoideae and Hemerocallidoideae. The core asparagoid and Xanthorrhoeaceae *s*.*l*. were sister (88/97/1.0) to Xeronemataceae alone. Collectively, this large clade was sister (87/97/1.0) to Iridaceae. The sister relationship between Ixioliriaceae and Tecophilaeaceae had strong support (86/96/1.0), but its position relative to Doryanthaceae remains unclear. However, a clade including Doryanthaceae, Ixioliriaceae, Tecophilaeaceae and the above-mentioned families was strongly supported (88/97/1.0). In turn, this clade was sister (60/< 50/1.0) to the astelioid clade that included Boryaceae, Blandfordiaceae, Asteliaceae, Lanariaceae and Hypoxidaceae. The monophyletic Orchidaceae was the first to diverge and was sister to all other asparagoids with high support (92/100/1.0).

### Divergence Time Estimation

The mean path lengths (MPL) clock tests [34] revealed significant deviations from clock-like behaviour at most nodes of the tree for Asparagales (clock tests: 265; accepted: 14; rejected: 251). Hence, we used BEAST [35], which implements a “relaxed clock” methodology that does not assume any correlation between rates (thus accounting for lineage-specific rate heterogeneity), to estimate ages and the phylogenetic tree simultaneously. At the same time, we also used PATHd8, with the mean path length method; this programme is faster for a large dataset and permits rate changes across the tree [34]. We obtained slight younger ages in the results using PATHd8 than using BEAST.

**Figure 3:**
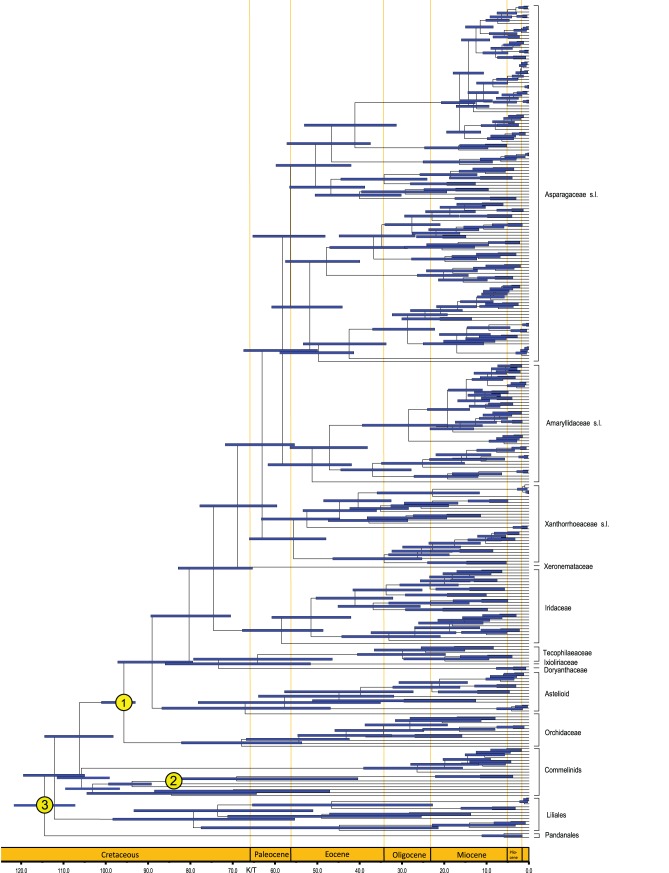
Divergence time estimates for Asparagales, based on four cpDNA genes (atpB, matK, ndhF and rbcL). The maximum clade credibility tree from the divergence times estimated with BEAST. The 95% highest posterior density (HPD) estimates for each well-supported clade are represented by bars. Numbers at nodes are fossil calibration points: 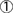 93 Ma, age for the most recent common ancestor (MRCA) of extant Asparagales; 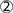 83.5 Ma, age for the MRCA of Zingiberales; 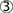 106.5±5.5 (93–120) Ma, age for the root of the tree (The upper age constraint of 120 Ma corresponds to the oldest known Monocot fossil). Detailed descriptions see the section of material and methods in text.

The BEAST analysis that treated fossil priors as lognormal distributions provided an older estimated age (102–143 Ma, data not presented) for crown group of Asparagales than that using an exponential distribution (93–101 Ma), as well as larger variances around age estimates, especially at the base of the tree (also see [36]). The topology showed good agreement with previous analyses of these data using Bayesian methods, with a few exceptions (Agavoideae, Scilloideae, Brodiaeoideae and Aphyllanthoideae present in some one clade but in different relatively position). The age estimates for crown and stem nodes are shown in Figure 3, with a chronogram calibrated against the geological timescale. Additional sampling and age estimates for families and subfamilies of Asparagales are summarised in Table 3.

**Table 3:** Sampling and age estimates for families and subfamilies of Asparagales.

Taxon	Number of species sampled	Crown node age (Ma)		Stem node age (Ma)	
		PATHd8	BEAST	PATHd8	BEAST
			Median (95% HPD)		Median (95% HPD)
Asparagaceae	122	36.4	56.4 (48.1–65.3)	40.6	58.3 (49.9–67.4)
-Nolinoideae	50	23.6	41.1 (31.3–53.1)	27.8	46.7 (37.4–57.3)
-Asparagoideae	5	9.6	16.4 (8.6–25.0)	27.8	46.7 (37.4–57.3)
-Lomandroideae	12	32.7	46.8 (38.8–56.6)	32.7	50.4 (42.0–59.8)
-Agavoideae	26	19.9	42.5 (33.8–53.3)	33.5	49.8 (41.4–58.9)
-Scilloideae	21	25.2	36.7 (28.7–47.1)	40.6	47.9 (40.0–57.6)
-Brodiaeoideae	7	25.1	20.2 (14.3–26.4)	40.5	47.9 (40.0–57.6)
-Aphyllanthoideae	1	n/a	n/a	40.5	49.8 (41.4–58.9)
Amaryllidaceae	41	30.1	51.2 (42.0–61.7)	41.6	58.3 (50.0–67.4)
-Amaryllidoideae	28	15.9	28.5 (19.2–39.4)	30.3	47.2 (38.1–56.5)
-Allioideae	12	30.3	37.0 (27.8–44.5)	30.3	47.2 (38.1–56.5)
-Agapanthoideae	1	n/a	n/a	33.7	51.2 (42.0–61.7)
Xanthorrhoeaceae	28	39.3	55.6 (48.0–66.1)	43.6	63.1 (55.4–71.8)
-Hemerocallidoideae	14	39.0	44.8 (36.0–53.4)	46.4	52.5 (44.7–63.2)
-Xanthorrhoeoideae	2	1.0	1.7 (0.3–3.8)	46.4	52.5 (44.7–63.2)
-Asphodeloideae	12	22.5	34.2 (25.3–46.4)	47.1	55.6 (48.0–66.1)
Xeronemataceae	1	n/a	n/a	55.8	68.9 (59.6–77.8)
Iridaceae	27	51.2	58.5 (48.6–67.7)	63.6	74.6 (65.3–82.9)
Tecophilaeaceae	6	20.4	29.9 (19.7–40.6)	34.1	64.1 (46.5–79.3)
Ixioliriaceae	1	n/a	n/a	34.1	64.1 (46.5–79.3)
Doryanthaceae	2	1.2	3.7 (0.7–7.7)	71.1	73.4(51.6–86.0)
Astelioid	15	65.2	67.1 (46.9–86.7)	85.1	89.1 (79.4–97.2)
Hypoxidaceae	8	15.6	22.9 (16.3–32.7)	37.6	39.8 (27.3–57.8)
Lanariaceae	2	n/a	n/a	38.3	39.8 (27.3–57.8)
Asteliaceae	1	32.6	29.5 (12.5–51.1)	37.6	44.9 (31.9–63.9)
Blandfordiaceae	3	2.1	4.1 (1.5–7.0)	38.3	57.7 (35.0–78.2)
Boryaceae	1	n/a	n/a	42.0	67.1 (46.9–86.7)
Orchidaceae	11	51.6	68.0 (53.7–82.1)	85.1	95.7 (93.0–101.0)
Commelinids	17	83.0	106.0 (98.8–113.1)	93.0	112.2 (105.0–120.0)
Liliales	12	54.8	79.5 (55.5–98.5)	95.1	106.2 (98.2–114.5)
Pandanales	2	n/a	5.9 (1.6–11.1)	120.0	114.5 (106.9–122.2)

## Discussion

### The Network Reveals a Useful Pattern in Asparagales

The detection of data conflicts and the extent to which data conflicts will affect the data analysis becomes an important first step in a phylogenetic analysis [6]. Phylogenetic networks, such as the split graphs produced by the neighbour-net algorithm, give a broad overview of competing evolutionary scenarios within a dataset [37]. These methods have been successfully used to analyse multigene plastid datasets (e.g. ferns, [38]; *Ranunculeae*, [39]), nuclear ribosomal DNA; *Acer*, [40]), and microbial and fungal evolution [9], [41], [42]. They have also been used in the context of genome sequencing surveys [43], [44]. However, the use of networks as a tool for large-scale phylogenetic research has rarely been reported in the scientific literature [6].

In this study, we used the phylogenetic network method neighbour net to analyse a larger-scale sampling datasets of Asparagales. The network tree summarised the majority data pattern in plastid sequences, which with long terminal edges clusters indicated strong support for the family system of Asparagales *sensu* APG III that was modified to include three expanded families [21], consistent with recently published analyses [14], [17], [22], [26]–[28], [45]. Most of the subfamilies (formerly as families) are pretty clear sustaining their taxonomic status in the split graphic. Otherwise, the short central edges forming the extensive cycles indicate broadly conflicting signals along the Asparagales backbone, but it is still clearly reflected in the underlying “skeleton” of evolutionary history. From the dominating tree-like pattern, we can anticipate that the four chloroplast genes in the data are compatible with one another and successfully infer phylogenetic trees [6].

The split pattern revealed strength of conflicting signals and helping us to understand how to affect the phylogenetic analysis. The phylogenetic indistinct taxon in regular phylogenetic analyses well appeared critical state on the split graph. In our case, at the base of Asparagales, astelioid, together with Orchidaceae, joined the main stem base of the network tree at the same position. However this situation means only included very little information about their relationships. It is perhaps unsurprising that the relationships of astelioid (especially Boryaceae) and Orchidaceae are unstable in some previous studies. For example, Boryaceae has sometimes been placed as sister to Orchidaceae (e.g. [11]), although with weak support, and there are other topologies, including one embedding Orchidaceae in a paraphyletic Boryaceae-Hypoxidaceae clade [32]. Unexpectedly, *M. biflora* complexly netted to Orchidaceae on network analyses (Figure 1), however this taxon has been grouped within Brodiaeoideae (Themidaceae sensu APG II) at present parsimony and Bayesian inference (Figure 2, part A) in line with previously reports [17], [22]. In case of sequencing or sampling errors, the split network is possibly more sensitive to exhibit artificial than regular phylogenetic analyses. The biased pattern of *M. biflora* suggests that resampling is necessary in order to find real situation.

The conflicting signals may be caused by homoplasy or stochastic noise rather than recombination that were not detected across the plastid genome in the core Asparagales [45]. DNA sequences from organellar genomes (e.g. mitochondria, plastids) are largely considered to be inherited uniparentally and non-recombining, with a single shared evolutionary history for the entire organellar genome [46]–[49]. Systematic mutational biases may also introduce conflicting phylogenetic signals within organelle sequences, especially between long-diverged taxa [50]. Although there may be reasons weak signals are introduced giving conflicting relationships, additional sequence data should allow identification of the bifurcating phylogenetic history of the organelle genome. Not unexpectedly, the continued examination of additional characters per taxon, 7 [17] and 17 plastid genes [23], and whole plastome sequences [45] gave higher resolution and bootstrap support to many clades in Asparagales.

Undoubtedly, it would be very wise to survey phylogenetic data using network methods before attempting to infer phylogenetic trees. Some attempts have begun [45], nevertheless the network methods should play a greater role in phylogenetic analyses than it has done to date. Compared with our inferred phylogenetic tree, it is worth noting that the network patterns reflect the tree bootstrap support to an extent, despite contrary opinions expressed previously [6], [51].

### Phylogeny of Asparagales

This study, with relatively dense taxon sampling and more diverse species representing more genera compared to previous phylogenetic studies, documented the stability of relationships within Asparagales. The family-level phylogenetic relationships found here were particularly congruent with other broad studies [14], [22], [23], [26]–[28], [45], indicating that the tree topologies in previous studies are robust with respect to the different samples used to represent genera and taxa sampled.

Relatively dense taxon sampling is generally a beneficial strategy for reducing long-branch attraction and obtaining more accurate inferences of phylogenetic relationships among and within large groups of organisms [52]–[55]. Long-branch attraction has been invoked for the placement of several problematic Asparagales taxa, such as Aphyllanthoideae and Ixioliriaceae, which are relatively isolated taxa with a long terminal branch. The position of *Aphyllanthes* in previous studies was labile and weakly supported [17], [22], [23]. In the neighbour-net tree in this study, *Aphyllanthes* had long edges that join to the base of Asparagaceae *s*.*l*., close to Lomandroideae, as has been found in other studies [17]. However, its position changed from sister to Agavoideae (Agavaceae *sensu* APG II) to sister to Brodiaeoideae (Themidaceae *sensu* APG II) in our MP and BI trees, respectively, but always formed a moderately to strongly supported group with Agavoideae, Scilloideae and Brodiaeoideae (63/91/1.0), which is also consistent with previous studies [22], [23], [26], [28]. Based on genome data (79-plastid gene matrix), Steele et al. [45] found that *Aphyllanthes* was sister to Agavoideae with moderate support and confirmed that it links the same subfamilies mentioned above using neighbour-net analyses. Obviously, *Aphyllanthes* may be suffering from not only long branch attraction (LBA), but also too few characters to define individual nearby branches as a result of rapid radiation [45].

Ixioliriaceae was inferred as a strongly supported sister group to Tecophilaeaceae in this study, a result that had variable support in previous analyses [17], [22], [26], [28]. Analyses of morphological data and base chromosome number support the sister relationship of these two families [56]. Doryanthaceae remain unresolved, forming either a polytomy or a weakly supported sister to the clade of Ixioliriaceae/Tecophilaeaceae and the remainder of Asparagales (except Astelioid and Orchidaceae), consistent with previous analyses [13], [26], [28].

Monophyly of the astelioid clade was well supported (83/91/1.0), including five small families (Boryaceae, Hypoxidaceae, Lanariaceae, Asteliaceae and Blandfordiaceae; Figure 2, part C), consistent with most previous studies [22], [23], [26], [28], [57], [58]. This clade has been demonstrated to have some shared morphological characters for all but Blandfordiaceae [57]. Little is gained by recognising the astelioid clade as a single family (Hypoxidaceae *s*.*l*.) to further reduce the number of families in Asparagales.

Our results highlight the largely robust framework for Asparagales, which is largely or completely congruent with the comparable taxonomic sampling in previous studies [14], [15], [17], [22], [23], [26]–[28], [45].

### Divergence Time Estimates

The age estimates obtained across the major clades of Asparagales from the PATHd8 and BEAST analyses compared here overlap considerably (see Table 3). Overall PATHd8 produced slightly younger ages than BEAST. The BEAST analyses that used multiple (three) constraints with exponential distribution may be a good alternative to a lognormal distribution in the face of inadequate palaeontological information [59], which yielded a narrower 95% higher posterior density (HPD) and generally younger node ages than the latter, as noted by Bell et al. [36].

We estimated that the stem group of Asparagales dates to ca. 99–113 Ma and that the crown group dates to ca. 93–101 Ma, which agrees reasonably with Bell et al. [36], who reported a crown age range of 83–103 Ma (see Appendix S15 in their paper). However, Janssen and Bremer [31] suggested somewhat older dates of ca. 122 Ma and ca. 119 Ma, respectively. The topology within Asparagales, especially near the base, in the latter differed substantially from our results; e.g. they did not place Orchidaceae as sister to the rest of the order. Comparable results in Magallón and Castillo [60] were ca. 133.1 (stem), 125 (crown), 118.6 (stem) and 112.6 (crown) Ma for relaxed and constrained penalised likelihood dating, respectively. These molecular-based estimates suggest a Cretaceous origin of Asparagales. In this study, the estimates are obviously close to the oldest known fossil record of Asparagales (93–105 Myr old, see [61] Supplementary Methods for details ).

Our estimated divergence time for the families in Asparagales is much younger than previously suggested by Janssen and Bremer [31], in which most families were indicated to be older than ca. 90 Ma. Orchidaceae is the largest and one of the ecologically and morphologically most diverse families of flowering plants [62]. Our results indicated that the most recent common ancestor of extant orchids lived in the Late Cretaceous (54–82 Ma), slightly overlapping the estimated age (76–84 Ma) based on the discovery of the first unambiguous fossil of Orchidaceae and a pollinator in amber [61]. Moreover, adding two newly described orchid fossils [63], Gustafsson et al. [64] reassessed the data and reported that all extant orchids shared a most recent common ancestor in the Late Cretaceous (ca. 77 Ma), suggesting that the diversification of orchids occurred in a period of global cooling after the early Eocene climatic optimum.

Iridaceae, with over 2,030 species in 65–75 genera, is the second largest family of Asparagales [65]. Based on plastid sequences and molecular clock techniques, Goldblatt et al. [65] inferred that Iridaceae diverged from the most closely related family, Doryanthaceae, ca. 82 Ma and that the crown group of the family diverged in the late Cretaceous ca. 66 Ma. The divergence of the stem group was dated to ca. 75 Ma and crown group to ca. 58 Ma. Goldblatt et al. [65] used a secondary date for the calibration point of the root node of Iridaceae, and this was suggested not to be ideal.

The split between core Asparagales and the remaining families is estimated after the K/T boundary. Furthermore, our molecular phylogenetic analyses suggest multiple rapid radiations have inferred throughout the diversification of major groups of Asparagales. For example, the largest orchid subfamilies diversification occur in a period of global cooling [64] and the possible radiation of lineages of Nolinoideae revealed from this study.

The fossil record of Asparagales is comparatively poor, with few fossils attributable to families reaching back beyond the Late Eocene, perhaps because of the herbaceous habit and widespread zoophilous pollination [66]. The use of more fossils with more sophisticated prior distribution affords exciting opportunities for divergence time estimation in the future. Despite various possible limitations, this analysis provided new insights into the diversification and the origin of the families in Asparagales.

## Materials and Methods

### Plant Materials

The taxa used for this study included 253 species of 201 genera representing all families in Asparagales [20]. In addition, 29 species representatives of Arecales, Zingiberales, Commelinales, Poales, Liliales and Pandanales were included, with two species of Pandanales as the nominated outgroup. The plant material used was either fresh or dried, collected from the field and dried, taken from specimens in herbaria, from the DNA Bank of the Royal Botanic Gardens, Kew (http://data.kew.org/dnabank/DnaBankForm.html) or the Medicinal Plant Resources Bank of the Korea National Research Resource Centre (KNRRC) at Gachon University (for details, see Table 1). All necessary permissions and approvals for the described plant and specimen sampling were obtained from the respective curators, i.e. RBG Kew Gardens (Dr. M. W. Chase), Kunming Botanic Garden (MOU), Ivana Franka Botanic Garden (MOU), Australia Royal Botanic Garden (MOU), KEW DNA Bank. Voucher specimens of the taxa were prepared; source, voucher information and database accession numbers are listed in Table 1.

**Table 1:** Vouchers with GenBank accession number for taxa included in this study.

Family/Tribe Taxa	Vouchers	sourcetype	Source(Institution)	Country	matK	rbcL	atpB	ndhF
Asparagales								
Higher asparagoids								
Asparagaceae								
Nolinoideae								
* Danae racemosa*	Chase 121	DNA	KEW DNABank	UK	KimJH,2010	KimJH,2010	JX903679	JX903260
* Ruscus aculeatus*	J.H. Kim s.n. 2008	Fresh	RBG Kew Garden	UK	KimJH,2010	KimJH,2010	JX903680	JX903261
* Ruscus streptophyllus*	Chase 21990	DNA	KEW DNABank	UK	KimJH,2010	KimJH,2010	JX903681	JX903262
* Semele androgyna*	Chase 997	DNA	KEW DNABank	UK	KimJH,2010	KimJH,2010	JX903682	JX903263
* Aspidistra elatior*	Z. Jang 4805	Specimen	KUN	China	KimJH,2010	KimJH,2010	JX903683	JX903264
* Aspidistra yingjiangensis*	D.K. Kim 08-200	Fresh	Kunming Botanic Garden	China	JX903532	JX903123	JX903684	JX903265
* Rohdea japonica*	D.K. Kim 05-005	Fresh	Kunming Botanic Garden	China	KimJH,2010	KimJH,2010	JX903685	JX903266
* Tupistra aurantiaca*	Chase 1100	DNA	KEW DNABank	UK	KimJH,2010	KimJH,2010	JX903686	JX903267
* Convallaria majalis*	D.K. Kim 04-082	Fresh	Field work	Korea	KimJH,2010	KimJH,2010	JX903687	JX903268
* Reineckea carnea*	Wu 454	DNA	KEW DNABank	UK	KimJH,2010	KimJH,2010	JX903688	JX903269
* Speirantha gardenii*	Chase 495	DNA	KEW DNABank	UK	KimJH,2010	KimJH,2010	JX903689	JX903270
* Theropogon pallidus*	Chase 2933	DNA	KEW DNABank	UK	KimJH,2010	KimJH,2010	JX903690	JX903271
* Comospermum yedoense*	Chase 833	DNA	KEW DNABank	UK	KimJH,2010	KimJH,2010	JX903784	JX903366
* Liriope platyphylla*	D.K. Kim 07-001	Fresh	Field work	Korea	KimJH,2010	KimJH,2010	JX903691	JX903272
* Liriope spicata*	D.K. Kim 07-002	Fresh	Field work	Korea	KimJH,2010	KimJH,2010	JX903692	JX903273
* Ophiopogon jaburan*	D.K. Kim 07-004	Fresh	Field work	Korea	KimJH,2010	KimJH,2010	JX903693	JX903274
* Ophiopogon japonicus*	D.K. Kim 07-003	Fresh	Field work	Korea	KimJH,2010	KimJH,2010	JX903694	JX903275
* Ophiopogon stenophyllus*	D.K. Kim 08-207	Fresh	Kunming Botanic Garden	China	JX903533	JX903124	JX903695	JX903276
* Peliosanthes sp.*	Chase 847	DNA	KEW DNABank	UK	JX903535	JX903126	JX903697	JX903278
* Peliosanthes teta ssp. humilis*	Malayisa FRI 39983	DNA	KEW DNABank	UK	JX903534	JX903125	JX903696	JX903277
* Disporopsis pernyi*	Chase 493	DNA	KEW DNABank	UK	KimJH,2010	KimJH,2010	JX903698	JX903279
* Disporopsis sp.*	D.K. Kim 05-003	Fresh	Kunming Botanic Garden	China	KimJH,2010	KimJH,2010	JX903699	JX903280
* Maianthemum bifolium*	D.K. Kim 04-182	Fresh	Field work	Korea	KimDK,2012	KimDK,2012	JX903700	JX903281
* Maianthemum dilatatum*	D.K. Kim 04-165	Fresh	Field work	Korea	KimJH,2010	KimJH,2010	JX903701	JX903282
* Maianthemum stellatum*	D.K. Kim 08-229	Fresh	RBG Kew Garden	UK	JX903536	JX903127	JX903702	JX903283
* Polygonatum desoulavyi*	D.K. Kim 09-225	Fresh	Field work	Korea	JX903537	JX903128	JX903703	JX903284
* Polygonatum falcatum*	D.K. Kim 09-191	Fresh	Field work	Korea	JX903538	JX903129	JX903704	JX903285
* Polygonatum humile*	D.K. Kim 04-029	Fresh	Field work	Korea	KimJH,2010	KimJH,2010	JX903705	JX903286
* Polygonatum inflatum*	D.K. Kim 04-043	Fresh	Field work	Korea	KimJH,2010	HM640456	JX903706	JX903287
* Polygonatum involucratum*	D.K. Kim 04-059	Fresh	Field work	Korea	KimJH,2010	HM640457	JX903707	JX903288
* Polygonatum lasianthum* var. *coreanum*	D.K. Kim 04-046	Fresh	Field work	Korea	KimJH,2010	HM640458	JX903708	JX903289
* Polygonatum odoratum* var. *pluriflorum*	D.K. Kim 04-067	Fresh	Field work	Korea	KimJH,2010	HM640459	JX903709	JX903290
* Polygonatum stenophyllum*	D.K. Kim 08-156	Fresh	Field work	Korea	KimDK,2012	KimDK,2012	JX903710	JX903291
*Maianthemum bicolor*	D.K. Kim 04-077	Fresh	Field work	Korea	KimJH,2010	KimJH,2010	JX903711	JX903292
* Maianthemum dahurica*	D.K. Kim 05-082	Fresh	Field work	Korea	KimJH,2010	KimJH,2010	JX903712	JX903293
* Maianthemum japonica*	D.K. Kim 04-039	Fresh	Field work	Korea	KimJH,2010	KimJH,2010	JX903713	JX903294
* Dracaena aubryana*	Chase 1102	DNA	KEW DNABank	UK	KimJH,2010	KimJH,2010	JX903714	JX903295
* Dracaena deremensis*	J.H. Kim 2009 s.n.	Fresh	Ivana Franka Boranic Garden	Ukraine	JX903539	*AB029848	JX903715	JX903296
* Dracaena hookeriana*	D.K. Kim 09-027	Fresh	Australia Royal Botanic Garden	Austalia	JX903540	*AM235113	JX903716	JX903297
* Dracaena schizantha*	Chase 21514	DNA	KEW DNABank	UK	KimJH,2010	KimJH,2010	JX903717	JX903298
* Pleomele javanica*	Chase 1240	DNA	KEW DNABank	UK	JX903541	JX903130	JX903718	JX903299
* Sansevieria trifasciata*	D.K. Kim 07-005	Fresh	Field work	Korea	KimJH,2010	KimJH,2010	JX903719	JX903300
* Beaucarnea recurvata*	D.K. Kim 09-002	Fresh	Field work	Korea	JX903542	JX903131	JX903723	JX903304
* Calibanus hookeri*	Chase 1006	DNA	KEW DNABank	UK	KimJH,2010	KimJH,2010	JX903724	JX903305
* Dasylirion wheeleri*	Chase 3469	DNA	KEW DNABank	UK	KimJH,2010	KimJH,2010	JX903725	JX903306
* Nolina bigelovii*	D.K. Kim 08-231	Fresh	RBG Kew Garden	UK	JX903543	JX903132	JX903726	JX903307
* Nolina recurvata*	Chase 3466	DNA	KEW DNABank	UK	KimJH,2010	KimJH,2010	JX903727	JX903308
* Eriospermum abyssinicum*	Chase 2051	DNA	KEW DNABank	UK	KimJH,2010	KimJH,2010	JX903720	JX903301
* Eriospermum cooperi* var. *natalensis*	Chase 2052	DNA	KEW DNABank	UK	KimJH,2010	KimJH,2010	JX903721	JX903302
* Eriospermum parvifolium*	Chase 2053	DNA	KEW DNABank	UK	KimJH,2010	KimJH,2010	JX903722	JX903303
*Asparagoideae*								
* Asparagus cochinchinensis*	D.K. Kim 04-122	Fresh	Field work	Korea	KimJH,2010	KimJH,2010	JX903789	JX903371
* Asparagus densiflorus*	D.K. Kim 08-198	Fresh	Kunming Botanic Garden	China	JX903580	JX903171	JX903790	JX903372
* Asparagus oligoclonos*	D.K. Kim 08-007	Fresh	Field work	Korea	KimDK,2012	KimDK,2012	JX903791	JX903373
* Asparagus schoberioides*	D.K. Kim 05-165	Fresh	Field work	Korea	KimJH,2010	KimJH,2010	JX903792	JX903374
* Hemiphylacus latifolius*	Chase 668	DNA	KEW DNABank	UK	KimJH,2010	KimJH,2010	JX903793	JX903375
Lomandroideae								
* Acanthocarpus preisii*	Chase 2228	DNA	KEW DNABank	UK	JX903591	JX903182	JX903820	JX903403
* Arthropodium cirratum*	Chase 651	DNA	KEW DNABank	UK	KimJH,2010	KimJH,2010	JX903821	JX903404
* Chamaexeros serra*	Brummitt 31374	DNA	KEW DNABank	UK	JX903593	JX903184	JX903823	JX903406
* Cordyline cannifolia*	Chase 17936	DNA	KEW DNABank	UK	JX903594	JX903185	JX903824	JX903407
* Cordyline pumilio*	Chase 14228	DNA	KEW DNABank	UK	JX903595	JX903186	JX903825	JX903408
* Laxmannia squarrosa*	Chase 2214	DNA	KEW DNABank	UK	KimJH,2010	KimJH,2010	JX903826	JX903409
* Lomandra hastilis*	Brummitt George & Oliver 21239	DNA	KEW DNABank	UK	KimJH,2010	KimJH,2010	JX903827	JX903410
*Lomandra longifolia*	D.K. Kim 09-038	Fresh	Field work	Korea	*DQ401356	JX903187	JX903828	JX903411
* Lomandra ordii*	Brummitt 21345	DNA	KEW DNABank	UK	JX903596	JX903188	JX903829	JX903412
*Sowerbaea juncea*	Chase 454	DNA	KEW DNABank	UK	JX903597	JX903189	JX903830	JX903413
* Thysanotus sp.*	Chase 2218	DNA	KEW DNABank	UK	JX903598	JX903190	JX903831	JX903414
* Trichopetalum plumosum*	Cult ADU ex 1135	DNA	KEW DNABank	UK	JX903599	JX903191	JX903832	JX903415
Agavoideae								
* Agave americana*	D.K. Kim 08-193	Fresh	Field work	Korea	JX903544	JX903133	JX903729	JX903310
* Agave ghiesbrechtii*	Chase 3467	DNA	KEW DNABank	UK	KimJH,2010	KimJH,2010	JX903730	JX903311
* Anemarrhena asphodeloides*	Kew 1156	DNA	KEW DNABank	UK	KimJH,2010	KimJH,2010	JX903778	JX903360
* Anthericum liliago*	Chase 515	DNA	KEW DNABank	UK	KimJH,2010	KimJH,2010	JX903779	JX903361
* Anthericum ramosum*	J.H. Kim 2009 s.n.	Fresh	Ivana Franka Boranic Garden	Ukraine	JX903578	JX903168	JX903780	JX903362
* Behnia reticulata*	Goldblatt 9273	DNA	KEW DNABank	UK	KimJH,2010	KimJH,2010	JX903794	JX903376
* Camassia cusickii*	Cronquist 6549	DNA	KEW DNABank	UK	KimJH,2010	KimJH,2010	JX903801	JX903383
* Chlorogalum pomeridianum*	Chase 838	DNA	KEW DNABank	UK	JX903545	JX903134	JX903731	JX903312
* Chlorophytum orchidistrum*	Chase 2155	DNA	KEW DNABank	UK	KimJH,2010	KimJH,2010	JX903781	JX903363
* Chlorophytum suffructicosum*	Chase 1043	DNA	KEW DNABank	UK	KimJH,2010	KimJH,2010	JX903782	JX903364
* Chlorophytum tetraphyllum*	Chase 1044	DNA	KEW DNABank	UK	KimJH,2010	JX903169	JX903783	JX903365
* Echeandia sp.*	Chase 602	DNA	KEW DNABank	UK	KimJH,2010	KimJH,2010	JX903785	JX903367
* Hagenbachia panamensis*	Correa et al. 2629 K (10/1978)	DNA	KEW DNABank	UK	JX903579	JX903170	JX903786	JX903368
* Herreria salsaparilha*	Chase 2154	DNA	KEW DNABank	UK	KimJH,2010	KimJH,2010	JX903795	JX903377
* Herreriopsis elegans*	Maurin & Rakotonasolo 90	DNA	KEW DNABank	UK	JX903581	JX903172	JX903796	JX903378
* Hesperocallis undulata*	Cranfill&Schmid s.n.	DNA	KEW DNABank	UK	KimJH,2010	KimJH,2010	JX903797	JX903379
* Hastingsia serpentinicola*	Hufford 817	DNA	KEW DNABank	UK	JX903586	JX903177	JX903807	JX903389
* Hosta capitata*	D.K. Kim 09-008	Fresh	Field work	Korea	KimDK,2012	KimDK,2012	JX903732	JX903313
* Hosta minor*	D.K. Kim 08-086	Fresh	Field work	Korea	KimDK,2012	KimDK,2012	JX903733	JX903314
* Hosta plantaginea*	Jin Xiow Feng s.n.	Fresh	Kunming Botanic Garden	China	KimJH,2010	KimJH,2010	JX903734	JX903315
* Hosta yingeri*	D.K. Kim 08-011	Fresh	Field work	Korea	KimDK,2012	KimDK,2012	JX903735	JX903316
* Leucocrinum montanum*	Chase 795	DNA	KEW DNABank	UK	KimJH,2010	KimJH,2010	JX903787	JX903369
* Paradisea liliastrum*	Chase 826	DNA	KEW DNABank	UK	KimJH,2010	KimJH,2010	JX903736	JX903317
* Paradisea minor*	D.B. Yang s.n.	Specimen	KUN	China	KimJH,2010	KimJH,2010	JX903737	JX903318
* Yucca filamentosa*	D.K. Kim 06-077	Fresh	Field work	Korea	KimJH,2010	KimJH,2010	JX903738	JX903319
* Yucca queretaroensis*	D.K. Kim 08-230	Fresh	Field work	Korea	JX903546	JX903135	JX903739	JX903320
Scilloideae								
* Bellevalia pycnantha*	Chase 21821	DNA	KEW DNABank	UK	JX903582	JX903173	JX903798	JX903380
* Bellevalia romana*	D.K. Kim 08-224	Fresh	Field work	Korea	JX903583	JX903174	JX903799	JX903381
* Bowiea volubilis*	Chase 176	DNA	KEW DNABank	UK	KimJH,2010	KimJH,2010	JX903800	JX903382
* Dipcadi filifolium*	Chase 1783	DNA	KEW DNABank	UK	KimJH,2010	KimJH,2010	JX903802	JX903384
* Drimia altissima*	Chase 1870	DNA	KEW DNABank	UK	KimJH,2010	KimJH,2010	JX903803	JX903385
* Drimiopsis maxima*	Chase 17509	DNA	KEW DNABank	UK	JX903584	JX903175	JX903804	JX903386
* Eucomis humilis*	Chase 1847	DNA	KEW DNABank	UK	KimJH,2010	KimJH,2010	JX903805	JX903387
* Eucomis punctata*	J.H. Kim 2009 s.n.	Fresh	Ivana Franka Boranic Garden	Ukraine	JX903585	JX903176	JX903806	JX903388
* Hyacinthella nervosa*	Chase 21826	DNA	KEW DNABank	UK	JX903587	JX903178	JX903808	JX903390
* Hyacinthoides hispanica*	Chase 16564	DNA	KEW DNABank	UK	JX903588	JX903179	JX903809	JX903391
* Lachenalia carnosa*	Chase 2261	DNA	KEW DNABank	UK	KimJH,2010	KimJH,2010	JX903810	JX903392
* Ledebouria cooperi*	Chase 1786	DNA	KEW DNABank	UK	KimJH,2010	KimJH,2010	JX903811	JX903393
* Massonia angustifolia*	Chase 5666	DNA	KEW DNABank	UK	KimJH,2010	KimJH,2010	JX903812	JX903394
* Merwilla aurea*	LHMS 2387	DNA	KEW DNABank	UK	JX903589	JX903180	JX903813	JX903395
* Muscari aucheri*	Chase 21845	DNA	KEW DNABank	UK	KimJH,2010	KimJH,2010	JX903814	JX903396
* Ornithogalum armeniacum*	Chase 1682	DNA	KEW DNABank	UK	KimJH,2010	KimJH,2010	*AF168935	JX903397
* Ornithogalum caudatum*	D.K. Kim 09-028	Fresh	Field work	Korea	JX903590	JX903181	JX903815	JX903398
* Ornithogalum shawii*	Chase 1012	DNA	KEW DNABank	UK	KimJH,2010	KimJH,2010	JX903816	JX903399
* Rhadamanthus convallarioides*	Goldblatt, 10852	DNA	KEW DNABank	UK	KimJH,2010	KimJH,2010	JX903817	JX903400
* Scilla scilloides*	D.K. Kim 05-039	Fresh	Field work	Korea	KimJH,2010	KimJH,2010	JX903818	JX903401
* Urginea epigea*	Chase 2055	DNA	KEW DNABank	UK	KimJH,2010	KimJH,2010	JX903819	JX903402
Brodiaeoideae								
* Bessera elegans*	Chase 626	DNA	KEW DNABank	UK	KimJH,2010	KimJH,2010	JX903833	JX903416
* Bloomeria crocea* var. *aurea*	Chase 1010	DNA	KEW DNABank	UK	KimJH,2010	KimJH,2010	JX903834	JX903417
* Dandya thadhowardii*	Chase S.N.	DNA	KEW DNABank	UK	KimJH,2010	KimJH,2010	JX903835	JX903418
* Dichelostemma multiflorum*	Chase 1830	DNA	KEW DNABank	UK	KimJH,2010	KimJH,2010	JX903836	JX903419
* Milla biflora*	Chase 1907	DNA	KEW DNABank	UK	HM640641	HM640523	JX903837	JX903420
* Muilla maritima*	Chase 779	DNA	KEW DNABank	UK	KimJH,2010	KimJH,2010	JX903838	JX903421
* Triteleia peduncularis*	Chase 1860	DNA	KEW DNABank	UK	KimJH,2010	KimJH,2010	JX903839	JX903422
Aphyllanthoideae								
* Aphyllanthes monspeliensis*	Chase 614	DNA	KEW DNABank	UK	KimJH,2010	KimDK,2012	JX903788	JX903370
Amaryllidaceae								
Amaryllidoideae								
* Amaryllis belladona*	KEW 612	DNA	KEW DNABank	UK	JX903555	JX903144	JX903750	JX903333
* Apodolirion cedarbergense*	Graham Duncan	DNA	KEW DNABank	UK	JX903556	JX903145	JX903751	JX903334
* Calostemma lutea*	Chase 1505	DNA	KEW DNABank	UK	JX903557	JX903146	JX903752	JX903335
* Clivia nobilis*	Chase 3080	DNA	KEW DNABank	UK	KimJH,2010	JX903147	JX903753	JX903336
* Crinum asiaticum* var. *japonicum*	K.H. Tae 2004 s.n.	DNA	KNRRC	Korea	KimJH,2010	KimJH,2010	JX903754	JX903337
* Cybistetes longifolia*	KEW 3643	DNA	KEW DNABank	UK	JX903558	JX903148	JX903755	JX903338
* Cyrtanthus purpureus*	Chase 1572	DNA	KEW DNABank	UK	JX903559	JX903149	JX903756	JX903339
* Eustephia darwinii*	Chase 559	DNA	KEW DNABank	UK	JX903560	JX903150	JX903757	JX903340
* Gethyllis brittoniana*	Van Jaarsveld 5618	DNA	KEW DNABank	UK	JX903561	JX903151	JX903758	JX903341
* Habranthus martinezii*	Chase 1023	DNA	KEW DNABank	UK	JX903562	JX903152	JX903759	JX903342
* Haemanthus albiflos*	Chase 17939	DNA	KEW DNABank	UK	JX903563	JX903153	JX903760	JX903343
* Hieronymiella var. latifolia*	Chase 1901	DNA	KEW DNABank	UK	JX903564	JX903154	JX903761	JX903344
* Hippeastrum psittacinum*	Chase 14823	DNA	KEW DNABank	UK	JX903565	JX903155	JX903762	JX903345
* Hymenocallis littoralis*	Chase 2027	DNA	KEW DNABank	UK	JX903566	JX903156	JX903763	JX903346
* Ismene longifolia*	Chase 3583	DNA	KEW DNABank	UK	JX903567	JX903157	JX903764	JX903347
* Leucojum roseum*	Chase 1524	DNA	KEW DNABank	UK	JX903568	JX903158	JX903765	JX903348
* Lycoris sanguinea* var. *koreana*	D.K. Kim 06-100	Fresh	Field work	Korea	KimDK,2012	KimDK,2012	JX903766	JX903349
* Lycoris uydoensis*	D.K. Kim 05-102	Fresh	Field work	Korea	KimJH,2010	KimJH,2010	JX903767	JX903350
* Narcissus tazetta* var. *chinensis*	D.K. Kim 06-167	Fresh	Field work	Korea	KimJH,2010	KimJH,2010	JX903768	JX903351
* Nerine alta*	Chase 18199	DNA	KEW DNABank	UK	JX903569	JX903159	JX903769	JX903352
* Pancratium canariense*	Chase 17733	DNA	KEW DNABank	UK	JX903570	JX903160	JX903770	JX903353
* Paramongaia weberbaueri*	Chase 1594	DNA	KEW DNABank	UK	JX903571	JX903161	JX903771	JX903354
* Scadoxus cinnabarinus*	Chase 549	DNA	KEW DNABank	UK	JX903572	JX903162	JX903772	JX903355
* Scadoxus puniceus*	D.K. Kim 09-011	Fresh	Field work	Korea	JX903573	JX903163	JX903773	JX903356
* Stenomesson miniatum*	Chase 16481	DNA	KEW DNABank	UK	JX903574	JX903164	JX903774	*FJ264208
* Ungernia flava*	Chase 3640	DNA	KEW DNABank	UK	JX903575	JX903165	JX903775	JX903357
* Vagaria parviflora*	Chase 1066	DNA	KEW DNABank	UK	JX903576	JX903166	JX903776	JX903358
* Zephyranthes simpsonii*	Chase 1839	DNA	KEW DNABank	UK	JX903577	JX903167	JX903777	JX903359
Allioideae								
* Allium microdictyon*	D.K. Kim 08-002	Fresh	Field work	Korea	KimDK,2012	KimDK,2012	JX903740	JX903321
* Allium ochotense*	D.K. Kim 04-142	Fresh	Field work	Korea	KimJH,2010	KimJH,2010	JX903741	JX903322
* Allium sacculiferum*	D.K. Kim 08-095	Fresh	Field work	Korea	KimDK,2012	KimDK,2012	*AF209525	JX903323
* Allium thunbergii*	D.K. Kim 08-220	Fresh	Field work	Korea	JX903547	JX903136	*AY147628	JX903324
* Ipheion uniflorum(uniflora)*	Chase 449	DNA	KEW DNABank	UK	KimJH,2010	KimJH,2010	JX903742	JX903325
* Leucocoryne pauciflora*	Chase 16462	DNA	KEW DNABank	UK	JX903548	JX903137	JX903743	JX903326
* Nothoscordum bivalve*	D.K. Kim 08-215	Fresh	Field work	Korea	JX903549	JX903138	JX903744	JX903327
* Nothoscordum borbonicum*	D.K. Kim 08-189	Fresh	Field work	Korea	JX903550	JX903139	JX903745	JX903328
* Nothoscordum texanum*	Chase 1593	DNA	KEW DNABank	UK	JX903551	JX903140	JX903746	JX903329
* Tristagma nivale*	Chase 2757	DNA	KEW DNABank	UK	JX903552	JX903141	JX903747	JX903330
* Tristagma uniflorum*	H. Murakami 631	Specimen	KYO	Japan	JX903553	JX903142	JX903748	JX903331
* Tulbaghia simmleri*	Chase 17513	DNA	KEW DNABank	UK	JX903554	JX903143	JX903749	JX903332
Agapanthoideae								
* Agapanthus africanus*	Chase 627	DNA	KEW DNABank	UK	KimJH,2010	KimJH,2010	JX903728	JX903309
Lower asparagoids								
Hemerocallidoideae								
* Caesia contorta*	Goldblatt 9406	DNA	KEW DNABank	UK	JX903610	JX903201	JX903858	JX903442
* Corynotheca micrantha*	Chase 2210	DNA	KEW DNABank	UK	JX903611	JX903202	JX903859	JX903443
* Chamaescilla sp.*	Chase 2208	DNA	KEW DNABank	UK	JX903592	JX903183	JX903822	JX903405
* Dianella ensifolia*	Akiyo Naiki 5510	Specimen	KUN	China	KimJH,2010	KimJH,2010	JX903860	JX903444
* Hemerocallis dumortieri*	D.K. Kim 08-145	Fresh	Field work	Korea	KimDK,2012	KimDK,2012	JX903861	JX903445
* Hemerocallis fulva*	D.K. Kim 08-152	Fresh	Field work	Korea	KimDK,2012	KimDK,2012	JX903862	JX903446
* Hemerocallis hongdoensis*	D.K. Kim 09-013	Fresh	Field work	Korea	JX903612	*AY149364	JX903863	JX903447
* Hemerocallis minor*	D.K. Kim 05-091	Fresh	Field work	Korea	KimJH,2010	KimJH,2010	JX903864	JX903448
* Johnsonia pubescens*	Chase 2213	DNA	KEW DNABank	UK	JX903613	JX903203	JX903865	JX903449
* Pasithea coerulea*	Chase 512	DNA	KEW DNABank	UK	JX903614	JX903204	JX903866	JX903450
* Phormium tenax*	Chase 177	DNA	KEW DNABank	UK	JX903615	JX903205	JX903867	JX903451
* Stawellia dimorphantha*	P.J. Rudall, s.n.	DNA	KEW DNABank	UK	JX903616	*Z77306	JX903868	*FJ707520
* Stypandra glauca*	Brummitt, George & Oliver 21223	DNA	KEW DNABank	UK	JX903617	JX903206	JX903869	JX903452
* Tricoryne elatior*	Chase 2219	DNA	KEW DNABank	UK	JX903618	JX903207	JX903870	JX903453
Xanthorrhoeoideae								
* Xanthorrhoea resinosa*	Chase 192	DNA	KEW DNABank	UK	KimJH,2010	KimJH,2010	JX903923	JX903504
* Xanthorrhoea media*	D.K. Kim 09-032	Fresh	Field work	Korea	JX903650	JX903234	JX903922	JX903503
Asphodeloideae								
* Aloe vera*					*AJ511390	*AJ512309	*AF168886	*AY225054
* Asphodeline lutea*	UCI Arb. 3440	DNA	KEW DNABank	UK	JX903600	JX903192	JX903840	JX903423
* Asphodelus aestivus*	Chase 482	DNA	KEW DNABank	UK	KimJH,2010	KimJH,2010	JX903841	JX903424
* Astroloba foliosa*	Chase 684	DNA	KEW DNABank	UK	JX903601	JX903193	JX903842	JX903425
* Bulbine semibarbata*	K. Dixon s.n.	DNA	KEW DNABank	UK	KimJH,2010	KimJH,2010	JX903843	JX903426
* Bulbinella cauda-felis*	UCI Arb. 359	DNA	KEW DNABank	UK	JX903602	JX903194	JX903844	JX903427
* Eremurus chinensis*	Qing 00317	DNA	KEW DNABank	UK	KimJH,2010	KimJH,2010	JX903845	JX903428
* Gasteria rawlinsoii*	Chase 18179	DNA	KEW DNABank	UK	JX903603	JX903195	JX903846	JX903429
* Haworthia coarctata*	Chase 3859	DNA	KEW DNABank	UK	JX903604	JX903196	JX903847	JX903430
* Kniphofia sp.*	D.K. Kim 08-187	Fresh	Field work	Korea	JX903605	*Z73689	*AJ417572	JX903431
* Poellnitzia rubiflora*	KEW 6534	DNA	KEW DNABank	UK	JX903606	JX903197	JX903848	JX903432
* Trachyandra esterhuysenae*	Fay s.n.	DNA	KEW DNABank	UK	JX903607	JX903198	JX903849	JX903433
Xeronemataceae								
* Xeronema callistemon*	Chase 653	DNA	KEW DNABank	UK	KimJH,2010	KimJH,2010	JX903924	JX903505
Iridaceae								
* Aristea monticala*	Compton 11967	DNA	KEW DNABank	UK	JX903622	JX903212	JX903878	JX903461
* Belamcanda chinensis*	D.K. Kim 08-186	Fresh	Field work	Korea	KimDK,2012	KimDK,2012	JX903879	JX903462
* Crocus banaticus*	D.K. Kim 09-004	Fresh	Field work	Korea	JX903623	JX903213	JX903880	JX903463
* Crocus cartwrighti*	Chase 11726	DNA	KEW DNABank	UK	JX903624	JX903214	JX903881	JX903464
* Dietes grandiflora*	D.K. Kim 09-021	Fresh	Field work	Korea	JX903625	JX903215	JX903882	JX903465
* Geissorhiza heterostyla*	Goldblatt & Manning 9668	DNA	KEW DNABank	UK	JX903626	JX903216	JX903883	JX903466
* Gladiolus illyricus*	Chase 9907	DNA	KEW DNABank	UK	JX903627	KimJH,2010	JX903884	JX903467
* Hermodactylus tuberosus*	Chase I-76	DNA	KEW DNABank	UK	JX903628	JX903217	JX903885	JX903468
* Iris confusa*	D.K. Kim 08-195	Fresh	Field work	Korea	JX903629	JX903218	JX903886	JX903469
* Iris minutiaurea*	D.K. Kim 08-124	Fresh	Field work	Korea	KimDK,2012	KimDK,2012	JX903887	JX903470
* Iris odaesanensis*	S.H. Park 2008 s.n.	Fresh	KRIBB	Korea	KimDK,2012	KimDK,2012	JX903888	JX903471
* Iris pseudoacorus*	D.K. Kim 09-055	Fresh	Field work	Korea	KimDK,2012	KimDK,2012	JX903889	JX903472
* Iris rossii*	D.K. Kim 05-048	Fresh	Field work	Korea	KimJH,2010	KimJH,2010	JX903890	JX903473
* Iris sanguinea*	D.K. Kim 08-056	Fresh	Field work	Korea	KimDK,2012	KimDK,2012	JX903891	JX903474
* Isophysis tasmanica*	J. Bruhl, TAS	DNA	KEW DNABank	UK	JX903630	JX903219	JX903892	JX903475
* Moraea riparia*	Goldblatt & Porter 12130	DNA	KEW DNABank	UK	JX903631	JX903220	JX903893	JX903476
* Neomarica northiana*	Solomon 6950	DNA	KEW DNABank	UK	JX903632	JX903221	JX903894	JX903477
* Nivenia stokoei*	KEW I-223	DNA	KEW DNABank	UK	JX903633	JX903222	JX903895	JX903478
* Pillansia templemanii*	Bean s.n.	DNA	KEW DNABank	UK	JX903634	JX903223	JX903896	JX903479
* Romulea bulbocodium*	Chase 21504	DNA	KEW DNABank	UK	JX903635	JX903224	JX903897	JX9034780
* Sisyrinchium palmifolium*	Chase 16458	DNA	KEW DNABank	UK	JX903636	JX903225	JX903898	JX9034781
* Solenomelus segethii*	Chase 19213	DNA	KEW DNABank	UK	JX903637	JX903226	JX903899	JX9034782
* Thereianthus racemosus*	KEW I-224	DNA	KEW DNABank	UK	JX903638	*AJ309663	JX903900	JX9034783
* Tigridia immaculata*	Rodríguez et al., 2832	DNA	KEW DNABank	UK	JX903639	JX903227	JX903901	JX9034784
* Trimezia martinicensis*	Chase 15941	DNA	KEW DNABank	UK	JX903640	JX903228	JX903902	JX9034785
* Watsonia anguta*	Goldblatt 6904	DNA	KEW DNABank	UK	JX903641	JX903229	JX903903	JX9034786
Tecophilaeaceae								
* Conanthera bifolia*	Chase 13821	DNA	KEW DNABank	UK	JX903646	JX903230	JX903916	JX903497
* Cyanella orchidiformis*	Chase 5896	DNA	KEW DNABank	UK	KimJH,2010	KimJH,2010	JX903917	JX903498
* Odontostomum hartwegii*	Chase 491	DNA	KEW DNABank	UK	JX903647	JX903231	JX903918	JX903499
* Tecophilaea cyanocrocus*	Chase 447	DNA	KEW DNABank	UK	KimJH,2010	KimJH,2010	JX903919	JX903500
* walleria gracilis*	Forest & Manning 542	DNA	KEW DNABank	UK	JX903648	JX903232	JX903920	JX903501
* Zephyra elegans*	Chase 1575	DNA	KEW DNABank	UK	JX903649	JX903233	JX903921	JX903502
Ixioliriaceae								
* Ixiolirion tataricum*	Chase 489	DNA	KEW DNABank	UK	KimJH,2010	KimJH,2010	JX903904	JX903487
Doryanthaceae								
* Doryanthes excelsa*	Chase 188	DNA	KEW DNABank	UK	KimJH,2010	KimJH,2010	JX903856	JX903440
* Doryanthes palmeri*	Chase 19153	DNA	KEW DNABank	UK	KimJH,2010	KimJH,2010	JX903857	JX903441
Astelioid								
Hypoxidaceae								
* Curculigo capitulata*	S.W. Lee 05-001	Fresh	Kunming Botanic Garden	China	KimJH,2010	KimJH,2010	JX903871	JX903454
* Hypoxis hemerocallidea*	Chase 3848	DNA	KEW DNABank	UK	KimJH,2010	KimJH,2010	JX903872	JX903455
* Hypoxis villosa*	D.K. Kim 09-025	Fresh	Field work	Korea	JX903619	JX903208	JX903873	JX903456
* Molineria capitulata*	Chase 1292	DNA	KEW DNABank	UK	AB088783	JX903209	JX903874	JX903457
* Pauridia longituba*	D. Snijman 1440 WBG	DNA	KEW DNABank	UK	JX903620	JX903210	JX903875	JX903458
* Rhodohypoxis baurii*	Chase 16460	DNA	KEW DNABank	UK	KimJH,2010	KimJH,2010	JX903876	JX903459
* Rhodohypoxis milloides*	Chase 479	DNA	KEW DNABank	UK	*AY368377	*Z77280	*AJ235582	*AY225062
* Spiloxene serrata*	Manning and Reeves JM&GR 2846	DNA	KEW DNABank	UK	JX903621	JX903211	JX903877	JX903460
Lanariaceae								
* Lanaria lanata*	Goldblatt & Manning 9410	DNA	KEW DNABank	UK	KimDK,2012	KimDK,2012	JX903905	JX903488
Asteliaceae								
* Astelia alpina*	Chase 1103	DNA	KEW DNABank	UK	KimJH,2010	KimJH,2010	JX903850	JX903434
* Milligania stylosa*	Chase 511	DNA	KEW DNABank	UK	KimJH,2010	KimJH,2010	JX903851	JX903435
Blandfordiaceae								
* Blandfordia cunninghamii*	R. Johnstone 2345 & A.E. Orme	DNA	KEW DNABank	UK	JX903608	JX903199	JX903852	JX903436
* Blandfordia grandiflora*	A.E. Orme 583 & S. Turrin	DNA	KEW DNABank	UK	JX903609	JX903200	JX903853	JX903437
* Blandfordia punicea*	Chase 519	DNA	KEW DNABank	UK	KimJH,2010	KimJH,2010	JX903854	JX903438
Boryaceae								
* Borya septentrionalis*	Chase 2205	DNA	KEW DNABank	UK	KimJH,2010	KimJH,2010	JX903855	JX903439
Orchidaceae								
* Apostasia wallichii*	Chase 15744	DNA	KEW DNABank	UK	JX903642	KimJH,2010	JX903906	JX903489
* Calanthe discolor*	D.K. Kim 05-035	Fresh	Field work	Korea	KimJH,2010	KimJH,2010	JX903907	JX903490
* Cephalanthera erecta*	D.K. Kim 08-048	Fresh	Field work	Korea	KimDK,2012	KimDK,2012	JX903908	JX903491
* Cephalanthera falcata*	D.K. Kim 08-110	Fresh	Field work	Korea	KimDK,2012	KimDK,2012	JX903909	JX903492
* Cephalanthera longibracteata*	D.K. Kim 05-016	Fresh	Field work	Korea	KimJH,2010	KimJH,2010	JX903910	JX903493
* Coelogyne sp.*	T.B. Tran T-37	Fresh	IEBR	Vietnam	JX903643	*AF074133	JX903911	*AY147777
*Cymbidium goeringii*	D.K. Kim 08-028	Fresh	Field work	Korea	KimDK,2012	KimDK,2012	JX903912	JX903494
* Cypripedium calceolus*	Chase 9484	DNA	KEW DNABank	UK	KimJH,2010	KimJH,2010	JX903913	JX903495
* Dendrobium acinaciforme*	T.B. Tran TN-32	Fresh	IEBR	Vietnam	JX903644	*FJ216578	JX903914	*U20534
* Epipactis thunbergii*	D.K. Kim 08-030	Fresh	Field work	Korea	JX903645	KimDK,2012	JX903915	JX903496
* Orchis rotundifolia*					*AY368385	*AY149368	*AY147623	*AY147783
Commelinids								
Commelinales								
Commelinaceae								
* Commelina communis*	D.K. Kim 07-006	Fresh	Field work	Korea	JX903665	JX903248	JX903938	JX903519
Arecales								
Araceae								
* Areca triandra*	AHBLoo 301	DNA	KEW DNABank	UK	*AM114664	JX903249	JX903939	*AY044535
* Arenga hastata*	Chase 18928	DNA	KEW DNABank	UK	JX903666	JX903250	JX903940	JX903520
* Astrocaryum mexicanum*	Chase 21299	DNA	KEW DNABank	UK	JX903667	JX903251	JX903941	JX903521
* Butia capitata*	Chase 21298	DNA	KEW DNABank	UK	JX903668	JX903252	JX903942	JX903522
* Calamus castaneus*	Baker 507	DNA	KEW DNABank	UK	JX903669	*M81810	JX903943	JX903523
* Nypa fruticans*	Chase 12603	DNA	KEW DNABank	UK	JX903670	JX903253	JX903944	JX903524
* Phoenix dactylifera*	Barrow 77	DNA	KEW DNABank	UK	JX903671	JX903254	JX903945	JX903525
* Ravenea sambiranensis*	Chase 18152	DNA	KEW DNABank	UK	JX903672	JX903255	JX903946	*EF128297
* Trachycarpus martianus*	Chase 30849	DNA	KEW DNABank	UK	JX903673	JX903256	JX903947	JX903526
Zingiberales								
Cannaceae								
* Canna indica*	D.K. Kim 08-190	Fresh	Field work	Korea	JX903674	JX903257	JX903948	JX903527
Costaceae								
* Costus woodsonii*	Chase 3911	DNA	KEW DNABank	UK	JX903675	*AF243510	JX903949	JX903528
Zingiberaceae								
* Roscoea cautleoides*	Chase 19223	DNA	KEW DNABank	UK	JX903676	JX903258	JX903950	JX903529
* Zingiber mioga*	D.K. Kim 08-069	Fresh	Field work	Korea	*GU180405	*AF243850	JX903951	JX903530
Poales								
Juncaceae								
* Juncus effusus*	D.K. Kim 09-078	Fresh	Field work	Korea	JX903677	*L12681	*AJ235509	*AF547015
Poaceae								
* Phragmites australis*					*AF144575	*U29900	*EF422973	*U21997
Typhaceae								
* Typha orienthalis*	D.K. Kim 09-011	Fresh	Field work	Korea	JX903678	JX903259	JX903952	JX903531
Liliales								
Colchicaceae								
* Disporum sessile*	D.K. Kim 04-076	Fresh	Field work	Korea	JX903651	JX903235	JX903925	JX903506
* Disporum smilacinum*	D.K. Kim 04-054	Fresh	Field work	Korea	JX903652	JX903236	JX903926	JX903507
* Disporum uniflorum*	D.K. Kim 04-089	Fresh	Field work	Korea	JX903653	JX903237	JX903927	JX903508
Liliaceae								
* Lilium distichum*	D.K. Kim 05-046	Fresh	Field work	Korea	JX903654	JX903238	JX903928	JX903509
* Lilium hansonii*	D.K. Kim 05-026	Fresh	Field work	Korea	JX903655	JX903239	JX903929	JX903510
* Lilium tsingtauense*	D.K. Kim 05-176	Fresh	Field work	Korea	JX903656	JX903240	JX903930	JX903511
Luzuriagaceae								
* Drymophila moorei*	R. Coveny et al., 6377	Fresh	Field work	Korea	JX903657	JX903241	JX903931	JX903512
Melanthiaceae								
* Chionographis japonica*	D.K. Kim 04-115	Fresh	Field work	Korea	JX903658	JX903242	JX903932	JX903513
* Heloniopsis orientalis*	D.K. Kim 06-058	Fresh	Field work	Korea	JX903659	JX903243	JX903933	JX903514
* Veratrum maackii* var. *japonicum*	D.K. Kim 06-129	Fresh	Field work	Korea	JX903660	JX903244	JX903934	JX903515
Smilacaceae								
* Smilax china*	D.K. Kim 04-096	Fresh	Field work	Korea	JX903661	JX903245	JX903935	JX903516
* Smilax riparia* var. *ussuriensis*	D.K. Kim 04-187	Fresh	Field work	Korea	JX903662	JX903246	JX903936	JX903517
Pandanales								
Pandanaceae								
* Pandanus veitchii*	J.H. Kim 2009 s.n.	Fresh	Ivana Franka Boranic Garden	Ukraine	JX903663	*AY952439	*AF168936	*AY191203
* Pandanus vandermeeschii*	Chase 15617	DNA	KEW DNABank	UK	JX903664	JX903247	JX903937	JX903518

Orders and families circumscriptions are as in APG III (2009) and Chase et al. (2009). The vouchers of all species studied were housed in source of institution.

KimJH, 2010: KIM, J. H., D. K. KIM, F. FOREST, M. F. FAY, AND M. W. CHASE. 2010. Molecular phylogenetics of Ruscaceae sensu lato and related families (Asparagales) based on plastid and nuclear DNA sequences. Annals of Botany 106: 775-790.

KimDK, 2012: KIM,D.K., J.S.Kim, J.H.Kim. 2012. The Phylogenetic Relationships of Asparagales in Korea Based on Five Plastid DNA Regions. Journal of Plant Biology 55: 325-341.

### DNA Extraction and Polymerase Chain Reaction Sequencing

Total genomic DNA was extracted from 0.5–1.0 g fresh or silica gel-dried leaves using the 2× CTAB buffer method [67]. Lipids were removed with SEVAG solution (24∶1 chloroform:isoamyl alcohol), and DNA was precipitated with isopropanol at –20°C. Total extracted DNA was dissolved in 1× TE buffer and stored at –70°C. The *atpB* gene was amplified using the primers and protocols of White et al. [68], Nickrent and Soltis [69] and Soltis and Soltis [70]. The *matK* gene was amplified with the primers and protocols of Johnson and Soltis [71] and Hilu et al. [25]; *ndhF* was amplified with the primers reported by Terry et al. [72] and Olmstead et al. [73]; and rbcL was amplified with the primers and protocols of Olmstead et al. [74], Shinwari et**al. [75] and Fay and Chase [76]. Amplifications were carried out in 50-µL reactions containing 2 units *Taq* DNA polymerase, 5 µL 10× reaction buffer (100 mM Tris-HCl, 500 mM KCl, 15 mM MgCl_2_), 2.5 mM dNTPs, and 5 pmol µL^–1^ forward and reverse primers using a Perkin-Elmer 9700 (Applied Biosystems, ABI, Beverly, MA, USA). Dimethyl sulphoxide (DMSO; 2%) was added to reduce the secondary structure in the polymerase chain reaction (PCR). PCR conditions consisted of an initial denaturation at 94°C for 2 min, followed by 30–35 cycles at 94°C for 1 min, 50°C–55°C for 1 min and 72°C for 3 min, followed by a final 7-min extension at 72°C. All PCR products were purified using ExoSAP-IT (USB Corporation, Cleveland, OH, USA), according to the manufacturer’s protocols. Dideoxy cycle sequencing was performed using the chain-termination method and an ABI Prism BigDye Reaction Kit (ver. 3.1) in accordance with the manufacturer’s protocols. Products were run on an ABI 3700 Genetic Analyser according to the manufacturer’s protocols. Sequence editing and assembly of contigs were carried out using the Sequence Navigator and AutoAssembler software (ABI).

### Sequence Alignment

All sequences were aligned initially in Muscal [77] and MacClade (ver. 4.0) [78] and then adjusted manually following the guidelines of Kelchner [79]. Manual alignment of *rbcL* and *atpB* was accomplished easily because no insertions/deletions occurred for *rbcL* and they were rare for *atpB*. In contrast, *matK* and *ndhF* were subject to length variation. These two genes were aligned and further edited manually by deleting small sections in which the homology of characters across taxa could not be determined with confidence. In total, the combined alignment was 6,699 characters in length (Table 2). The aligned matrix has been submitted as Appendix S1.

### Neighbour Net

Neighbour nets have the attractive property of always being represented in the plane through a circular ordering of the taxa. Although closely related to split decomposition [80], for larger datasets, the neighbour-net method often provides better resolution than split decomposition due to the criterion used to calculate support for relationships among taxa [9]. To construct neighbour nets, the default settings in SplitsTree4 [81] were used, applying uncorrected *P* distances with gaps and ambiguous sites coded as missing data. Bootstrap support for internal splits, which define clusters, was calculated with 1,000 replicates.

### Parsimony Analysis

PAUP* ver. 4.10b for Macintosh [82] was used for parsimony analysis. Tree searches were conducted using the Fitch (equal weight, EW) [83] criterion with 1,000 random sequence additions and tree bisection/reconnection (TBR) branch swapping, but permitting only five trees to be held at each step to reduce the time spent searching suboptimal “islands” of trees. All shortest trees collected in the 1,000 replicates were swapped on to completion without a tree limit. DELTRAN character optimisation was used to illustrate branch length throughout. To evaluate internal support, 1,000 bootstrap replicates were conducted with equal weights (EW) and successive approximation weights (SW; [84]), and TBR branch swapping with five trees held at each step and simple taxon addition [85]. The following descriptions for categories of bootstrap percentages were used: weak, ≤ 74; moderate, 75–84; well supported, 85–100 [14].

### Bayesian Analysis

Further phylogenetic analyses were performed using BI as implemented in MrBayes ver. 3.12 [86]. PAUP* ver. 4.10b and MrModeltest ver. 2.2 [87] were used to determine the best model of DNA substitution for each partition by evaluating all models against defaults of the programme. The GTR+*I*+*G* model (a general time-reversible model with a proportion of invariable sites and a gamma-shaped distribution of rates across sites) was chosen as the best-fit substitution model in all four partions. Consequently, the combined data matrix was assigned a model of six substitution types (*n* = 6) with a proportion of invariable sites. Four simultaneous Markov chain Monte Carlo (MCMC) chains were run for 1×10^7^ generations and sampled every 1,000 generations, and the first 25% sampled trees were excluded as burn-in. Post-burn-in samples of trees were used to construct a 50% majority rule consensus cladogram in PAUP* ver. 4.10b. The proportions of bifurcations found in this consensus tree are given as posterior clade probabilities (PPs). Bayesian analysis was performed twice to ensure convergence of the results.

### Molecular Dating and Fossil Calibration

We used the combined dataset to estimate the age of origin of Asparagales using the programmes PATHd8 [34] and BEAST v1.7.4 [35], [88]. The phylogenetic trees were constructed using MP with PAUP*4.0. The branch lengths on this tree were estimated using DELTRAN optimisation. We used the mean path length method of the PATHd8 programme. The MPL clock tests were used to test the molecular clock. The PATHd8 programme requires at least one reference point to be fixed. We used the oldest monocot fossil estimate of 120 Ma [89] as the fixed crown age of the root to calibrate the clock. BEAST v1.7.4 was also used to estimate the divergence times using multiple calibration points and a relaxed molecular clock approach. The BEAUti interface was used to create input files for BEAST with the tree priors set as follows: 1) age for the most recent common ancestor (MRCA) of extant Asparagales: exponential distribution with a mean of 2.0 and an offset 93 Ma that equalled the minimum age of the fossil (see discussion in [61], labelled 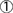 in Figure 3); 2) age for the MRCA of Zingiberales: exponential distribution with a mean of 2.0 and an offset 83.5 Ma which equalled the minimum age of the fossil (see [36], [90], labelled 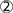 in Figure 3); 3) age for the root of the tree (The upper age constraint of 120 Ma for the calibrations above corresponds to the oldest known Monocot fossil [89]): normal prior distribution with mean 106.5 Ma and standard deviation of 5.5 (giving a 95% CI ranging from 93–120 Ma, labelled 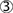 in Figure 3).

The general time-reversible (GTR+ *I*+*G*) nucleotide-substitution model was used for the molecular clock model and Yule Process was chosen as speciation process for data set. Several short BEAST runs were first performed to examine the performance of the MCMC. After optimal operator adjustment, as suggested by the output diagnostics, three final BEAST runs each containing 10,000,000 generations were performed, and a tree was saved every 1,000 generations. All resulting trees were then combined with LogCombiner v1.7.4 [35], with a burn-in of ca. 45%. Log files were analysed with Tracer v1.5 [91], to assess convergence and confirm that the combined effective sample sizes for all parameters were enough. A maximum credibility tree was then produced using TreeAnnotator v1.7.4 [35], [88]. These were visualised using FigTree v.1.3.1 with means and 95% HPDs of age estimates. An XML file for analyses has been submitted as Appendix S2.

## Supporting Information

Appendix S1 **The aligned data matrix in this study (Nexus).**
(NEX)

Appendix S2 **The XML file used for divergence time estimates in BEAST analysis.**
(XML)
